# Regulation of Autoimmune Germinal Center Reactions in Lupus-Prone BXD2 Mice by Follicular Helper T Cells

**DOI:** 10.1371/journal.pone.0120294

**Published:** 2015-03-13

**Authors:** Young Uk Kim, Hoyong Lim, Ha Eun Jung, Rick A. Wetsel, Yeonseok Chung

**Affiliations:** 1 Center for Immunology and Autoimmune Diseases, Institute of Molecular Medicine, the University of Texas Medical School at Houston, Houston, Texas, United States of America; 2 Graduate School of Biomedical Sciences, the University of Texas Health Science Center at Houston, Houston, Texas, United States of America; 3 Laboratory of Immunobiology, Research Institute of Pharmaceutical Sciences, College of Pharmacy, Seoul National University, Seoul, Republic of Korea; 4 Division of AIDS, Korea National Institute of Health, Cheongwon, Chungbuk, Republic of Korea; COCHIN INSTITUTE, Institut National de la Santé et de la Recherche Médicale, FRANCE

## Abstract

BXD2 mice spontaneously develop autoantibodies and subsequent glomerulonephritis, offering a useful animal model to study autoimmune lupus. Although initial studies showed a critical contribution of IL-17 and Th17 cells in mediating autoimmune B cell responses in BXD2 mice, the role of follicular helper T (Tfh) cells remains incompletely understood. We found that both the frequency of Th17 cells and the levels of IL-17 in circulation in BXD2 mice were comparable to those of wild-type. By contrast, the frequency of PD-1^+^CXCR5^+^ Tfh cells was significantly increased in BXD2 mice compared with wild-type mice, while the frequency of PD-1^+^CXCR5^+^Foxp3^+^ follicular regulatory T (Tfr) cells was reduced in the former group. The frequency of Tfh cells rather than that of Th17 cells was positively correlated with the frequency of germinal center B cells as well as the levels of autoantibodies to dsDNA. More importantly, CXCR5^+^ CD4^+^ T cells isolated from BXD2 mice induced the production of IgG from naïve B cells in an IL-21-dependent manner, while CCR6^+^ CD4^+^ T cells failed to do so. These results together demonstrate that Tfh cells rather than Th17 cells contribute to the autoimmune germinal center reactions in BXD2 mice.

## Introduction

CD4^+^ T cells provide ‘help’ to B cells by inducing somatic hypermutation, class-switching and the differentiation into memory B cells or long-lived plasma cells (PC) during germinal center (GC) reactions [1]. CXCR5^+^ICOS^+^PD-1^+^ follicular T helper (Tfh) cells have recently been shown to play crucial roles in promoting GC reactions [2] by providing IL-21and ICOS co-stimulation which are important for the above described germinal center B cell responses, as well as for the clonal expansion of antigen-specific B cells [3,4,5,6,7,8,9]. Therefore, Tfh cell responses are essential for the generation of effective humoral responses against invasion of infectious agents. By contrast, excessive Tfh cell responses to self-antigens are shown to be associated with antibody–mediated autoimmune diseases, such as systemic lupus erythematosus (SLE), rheumatoid arthritis (RA), Sjӧgren syndrome, and juvenile dermatomyositis [10,11,12,13,14]. Recent studies by our own lab and others uncovered the existence of a novel subset of regulatory T cells (Tfr) specialized for the regulation of germinal center reactions [15,16,17]. These cells express Foxp3, Bcl-6 and other surface markers such as CXCR5, PD-1 and ICOS, allowing them to efficiently migrate into B cell follicles where they can directly interact with Tfh cells and B cells. The importance of Tfr cells in regulating autoimmune humoral responses remains to be determined.

Multiple animal models of experimental autoimmune lupus and arthritis have been developed to study the pathophysiology of antibody-mediated autoimmunity. For instance, MRL^lpr/lpr^ mice spontaneously develop autoimmune lupus and arthritis; however, these mice are deficient in Fas [18], which is not common in patients with lupus. NZB/W F1 mice also spontaneously develop autoimmune lupus phenotypes but do not develop arthritis symptoms. In these aspects, BXD2 mice offer a novel animal model to study complex features of antibody-mediated autoimmune diseases. BXD2 is a recombinant inbred strain established by intercrossing a F2 of C57BL/6 and DBA/2J strains for more than 20 generations [19,20]. BXD2 mice spontaneously develop both autoimmune lupus symptoms including glomerulonephritis and also develop erosive arthritis, due to the excessive production of rheumatoid factor and autoantibodies [21,22,23,24]. By genetic linkage analysis, BXD2 mice have been shown to have several autoimmune loci such as *Lbw*, *Sle*, *Sles*, *Lmb* and *Asm2* as a result of complicated interaction of multiple genes from the original parental B6 and DBA/2 mice [21]. In addition, CD4^+^ T cells of BXD2 mice exhibit increased expression of CD28 which can further induce the expansion of CD86^+^ germinal center B cells and activation-induced cytidine deaminase (AID) expression in B cells [24]. These multiple genetic and immunologic features together seem to promote spontaneous autoimmune phenotypes in BXD2 mice. Since neither C57BL/6 mice nor DBA/2J mice spontaneously develop autoimmune lupus, the BXD2 strain offers a unique tool to study the mechanism of naturally occurring autoimmune B cell responses without any artificial genetic manipulation.

Of note, accumulating evidence suggests that IL-17 and IL-17-producing T helper (Th17) cells might also provide B cell help during GC reactions [25,26,27,28,29,30,31]. A series of studies have demonstrated that Th17 cells reside in the spontaneous GCs and that IL-17 enhances the formation of GCs in the BXD2 mice [23,30]. By inducing the expression of regulator of G-protein signaling proteins on B cells, IL-17 is known to stabilize the interaction of the germinal center B cells with nearby T cells [30,32]. In addition, IL-17 does not affect total number of Tfh cells and their function *in vitro*; however, IL-17RA deficient Tfh cells in BXD2 mice are shown to fail to localize in the GC light zone [31]. Although the Th17-related phenotypes of BXD2 mice have been well characterized, the contribution of Tfh cells to auto-reactive B cell responses in this strain has been incompletely understood. In the present study, we sought to determine the relative contribution of Th17 and Tfh cells to the autoimmunity in BXD2 mice by employing multiple *in vivo* and *in vitro* approaches. Our results strongly suggest that Tfh cell responses rather than Th17 cells are responsible for the auto-reactive GC reactions in BXD2 mice.

## Materials and Methods

### Mice

C57BL/6 and BXD2 mice were purchased from Jackson Laboratories (Bar Harbor, ME). All mice were maintained in the specific pathogen free facilities at the vivarium of the Institute of Molecular Medicine, the University of Texas Health Science Center at Houston and the Institute of Laboratory Animal Resources, Seoul National University. All animal experiments were performed using protocols approved by Institutional Animal Care and Use Committee of the University of Texas at Houston and Seoul National University.

### Flow cytometry

For cell phenotype analysis, lymphoid cells isolated from mouse spleens, peripheral lymph nodes, and Peyer’s patches were obtained and stained with PerCp-Cy5.5-conjugated anti-CD4 (clone GK1.5, BioLegend, San Diego, CA), APC-conjugated anti-CD45R/B220 (clone RA3–6B2, BioLegend), PE-conjugated anti-CD138 (clone 281–2, BioLegend), Alexa488 anti-GL7 (clone GL7, BD Pharmingen, San Jose, CA), PE-conjugated anti-CD95 (Fas, clone 15A7, eBioscience, San Diego, CA), FITC-conjugated anti-CD279 (PD-1, clone J43, eBioscience), Brilliant-Violet-421-conjugated anti-CD279 (clone 29F.1A12, BioLegend), PE-conjugated anti-CD184 (CXCR4, clone L276F12, BioLegend), Biotin-conjugated anti-CD185 (CXCR5, clone L138D7, BioLegend), PE or APC-conjugated Streptavidin (BioLegend), Alexa647-conjugated CD196 (CCR6, clone 140706, BD Pharmingen).

For intracellular cytokine staining, lymphoid cells isolated from mouse spleens, peripheral lymph nodes, and Peyer’s patches were stimulated with PMA (100 ng/ml, Sigma-Aldrich) and ionomycin (1 μM, Sigma-Aldrich) in the presence of Brefeldin A and Monensin (all from eBioscience) for 4 hours, followed by staining with PerCp-Cy5.5-conjugated anti-CD4 [33]. The cells were then resuspended in permeabilization buffer (eBioscience) for 30 min at 4°C, followed by staining with PE-conjugated anti-IL-17A (clone TC11–18H10.1, BioLegend), Alexa488-conjugated anti-IFN-γ (clone XMG1.2, eBioscience). For IL-21 staining, after permeabilization, cells were stained with Alexa488-conjugated anti-IL-17A (clone TC11–18H10.1, BioLegend), recombinant mouse IL-21R-Fc Chimera (R&D Systems), followed by APC or PE-conjugated human Fc IgG (R&D Systems, R&D Systems, Minneapolis, MN). For Foxp3^+^ cell detection, after surface staining, cells were incubated in Foxp3 staining buffer (eBioscience) for 30 min, followed by Alexa488-conjugated anti-Foxp3 (clone 150D/E4, eBioscienece). The stained cells were analyzed by FACSAria II flow cytometer (BD Bioscience, San Jose, CA), and the data were analyzed using FlowJo software (TreeStar, Ashland, OR).

### Immunohistochemical Analysis

Mouse spleens were fixed in 4% paraformaldehyde embedded in Tissue-Tek O.C.T compound (Sakura Finetek, Torrance, CA), and cut into 6 μm sections. The slides were incubated for 30 min with Image-iT FX Signal Enhancer (Life Technologies). After washing, slides were incubated with BlockAid Blocking Solution (Life Technologies) for 30 min, and then stained with Biotinylated Peanut Agglutinin (PNA, Vector Laboratories, Burlingame, CA), and hamster anti-mouse CD3e (clone 145–2C11, BioXcell, West Lebanon, NH). The slides were further incubated with Pacific Blue-conjugated anti-IgD (clone, 11–26c.2a, BioLegend), DyLight649-conjugated anti-Hamster IgG (clone Poly4055, BioLegend), and DyLight594-conjugated Streptavidin (Pierce Biotechnology, Rockford, IL). The slides were mounted with Fluoromount-G (SouthernBiotech, Birmingham, AB). Images were acquired with a Nikon TE2000E fluorescence microscope equipped with a Photometrics Coolsnap HQ2 camera.

### In vitro co-culture assay

Total B cells and CD4^+^ T cells were obtained by using anti-CD45R and anti-CD4 MACS columns (Miltenyi biotech, San Diego, CA). B220^+^GL7^-^IgD^+^ Naïve B cells, CD4^+^CXCR5^+^ and CD4^+^CCR6^+^ T cells from BXD2 mouse spleen were sorted by the FACSAria II (BD Biosciences). For *in vitro* antibody production assay, sorted 1 × 10^5^ Naïve B cells and diverse ratio of CXCR5^+^ or CCR6^+^ CD4 T cells or *in vitro* differentiated Th17 cells were co-cultured in the presence of 2 μg/ml soluble anti-CD3e (clone 145–2C11, BioXcell) and anti-IgM (AffiniPure F(ab’)_2_ Fragment Goat anti-IgM, μ chain specific, Jackson ImmunoResearch) for 7 days. For *In vitro* Th17 cell differentiation, sorted CD4^+^CD25^-^CD44^-^CD62L^+^ naïve T cells from BXD2 mice were stimulated for 5 days with plate-bound of anti-CD3(1 μg/ml) and anti-CD28 (1 μg/ml), in the presence of 2 ng/ml recombinant human TGF-β, 10 ng/ml recombinant mouse IL-6 (all purchased from Peprotech, Rocky Hill, NJ), 40 ng/ml recombinant mouse IL-23 (R&D systems), 10 μg/ml anti-mouse IFN-γ (clone XMG 1.2, BioXcell) and 10 μg/ml anti-mouse IL-4 (clone 11B11, BioXcell). For cytokine blockade, 10 μg/ml of recombinant human Fc-G1 (Human Fc-G1, BioXCell), anti-IL-17A (clone 50104, R&D Systems) or recombinant mouse IL-21R-Fc Chimera (R&D Systems) was added into the B and T cell co-culture every other day. Seven days later, the levels of murine IgG in the culture supernatant were determined by enzyme-linked immunosorbent assay (ELISA). For B cell proliferation assay, sorted 1 × 10^5^ naïve B cells labeled with CFSE (carboxyfluorescein diacetate succinimidyl ester, Invitrogen) were co-cultured with 5 × 10^4^ CXCR5^+^ or CCR6^+^ CD4 T cells as described above. The percent of divided cells was determined by flow cytometry.

### ELISA

To measure the levels of antibodies to dsDNA and histone in circulation, serum was collected from the wild type or BXD2 mice at various time points and was measured by ELISA as described previously [21]. Briefly, ELISA plates (Greiner Bio-one, Germany) were coated with 0.01% of poly-L-lysine (Sigma-Aldrich) for 1 hour prior to coating with 5 μg/ml of calf thymus dsDNA (Sigma-Aldrich) and 5 μg/ml histone (from calf thymus, Sigma-Aldrich) overnight at 37°C. The plates were blocked for 2 hours with PBS-Tween 20 (PBST) plus 3% milk. Sera were diluted in PBS with 1% of FBS, were transferred to the plates and were incubated for 2 hours at room temperature. Then the assay was performed with HRP-conjugated total IgG detection antibody (Goat anti-mouse IgG, SouthernBiotech). To measure the levels of IgG produced by B cells *in vitro*, total murine IgG was quantified in culture supernatants with a total IgG capture antibody (Donkey anti-mouse IgG (H+L), Jackson ImmunoResearch, West Grove, PA) and HRP-conjugated total IgG detection antibody.

### Quantitative real-time RT-PCR

Total RNA was extracted from splenocytes (1×10^6^ cells) or *in vitro* co-cultured cells with TRIzol (Invitrogen) and reverse transcribed using amfiRivert reverse transcriptase (GenDepot, Baker, TX) according to the manufacturer’s protocol. Gene expression was measured with iTaq-SYBR Green Supermix (Bio-Rad Laboratories, Hercules, CA) and the ABI-PRISM 7900 detection system (Applied Biosystems, Foster City, CA). Data were normalized to expression of the β-actin gene. The primer pairs used in quantitative RT-PCR are described in S1 Table.

### Statistical analysis

Data were analyzed with GraphPad Prism 5 (GraphPad, La Jolla, CA). Statistics were calculated with the two-tailed Student’s t-test. For correlative analyses among the percent of GL7^+^Fas^+^ germinal center B cells, the percent of T helper cell subsets, the levels of dsDNA specific autoantibodies, linear-regression analysis was performed. p- values below 0.05 were considered statistically significant.

## Results

### Spontaneous germinal center reactions in BXD2 mice

**Fig 1:**
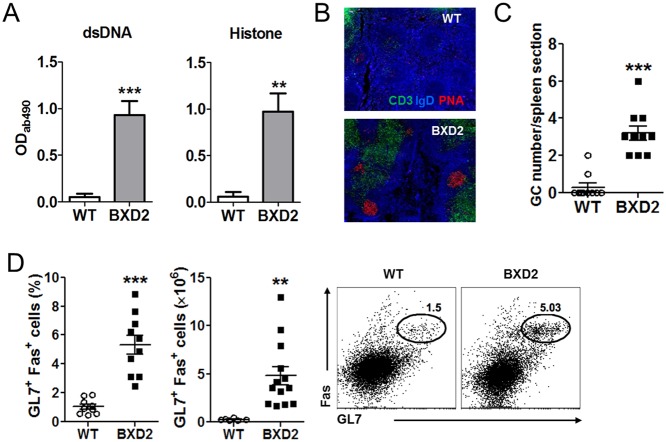
Spontaneous germinal center responses in BXD2 mice. (**A**) Auto-reactive autoantibody levels against double-strand DNA and histone in the sera of WT and BXD2 mice at the age of 6 months or older were measured by ELISA. (**B**) Immunofluorescence imaging of PNA^+^ (red) germinal center area of the spleen from WT control or BXD2 mice (×4 magnification). (**C**) The number of GC per spleen section in the WT and BXD2 mice was enumerated by fluorescence microscopy. (**D**) Flow cytometry analysis of the percentage and number of GL7^+^Fas^+^ germinal center B cells in the WT and BXD2 mice at the age of 6 months or older. Cells were gated on B220^+^ B cells. Data are represented as mean ± SEM. *p < 0.05, **p < 0.01, ***p < 0.001.

As a first step to determine the contribution of each T helper (Th) cell response to autoimmune lupus in BXD2 mice, we comparatively analyzed the production of autoantibodies and the generation of spontaneous germinal center reactions between BXD2 mice and C57BL/6 (wild-type) control mice at the age of 6 months or older. Consistent with previous reports [21,23,30,31,34], we observed that the levels of autoantibodies to double-stranded DNA and to histone in the sera of BXD2 mice were significantly higher than those in control mice (Fig. 1A). Similarly, we also observed spontaneous generation of GCs in the BXD2 mice (Fig. 1B and 1C), which was associated with increased frequency and number of GL7^+^Fas^+^ germinal center B cells in the spleens (Fig. 1D). Increased autoantibodies and spontaneous GCs were observed as early as 3 months of age in the BXD2 mice (data not shown). However, the frequency of GL7^+^Fas^+^ germinal center B cells remained comparable between the two groups in other secondary lymphoid organs, such as peripheral lymph nodes and Peyer’s patches (S1A Fig).

### Analysis of helper T cell subsets in BXD2 mice

**Fig 2:**
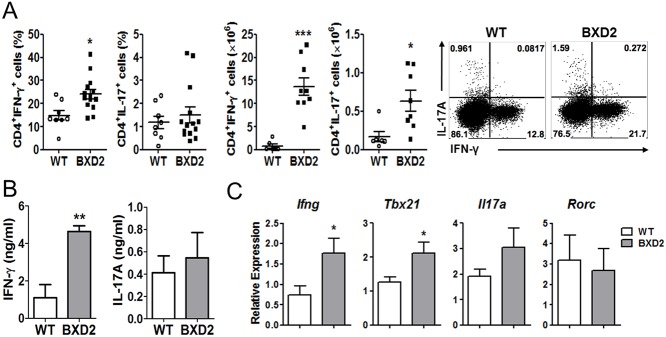
Th17 cells are not a major subset of T helper cells in BXD2 mice. (A) Flow cytometry analysis of the percentage and number of IFN-γ or IL-17A positive CD4+ T cells in the spleen from WT and BXD2 mice at the age of 6 months or older. (B) IL-17A levels in the sera of WT and BXD2 mice at the age of 6 months or older were measured by ELISA. (C) Quantitative RT-PCR analysis of Th1 or Th17 cell related genes from WT and BXD2 mice splenocytes at the age of 6 months or older. Data are representative of the analysis (A~C) indicate mean ± SEM. *p < 0.05, **p < 0.01, ***p < 0.001 compared with BXD2-WT mice.

We next analyzed each helper T cell subset in the spleens of BXD2 mice. The frequency of IFN-γ-producing Th1 cells was slightly but significantly higher in the spleens of BXD2 mice compared to control mice (Fig. 2A). In addition, due to increased cellularity in the spleens of BXD2 mice, the absolute number of Th1 cells was remarkably higher in this group. The proportion of Th1 cells were increased in most of the secondary lymphoid organs of BXD2 mice, except Peyer’s patches (S1A Fig). However, unlike previous study [30], we observed that the frequency of IL-17-producing Th17 cells in the spleens of BXD2 mice was comparable to that of control mice, although the absolute number of Th17 cells appeared to be increased in the former group (Fig. 2A). Similarly, the frequency of IL-17 secreting cells in total splenocytes of BXD2 mice was comparable to that of control mice, ruling out any increased production of IL-17 from non-CD4^+^ T cell population (S1B Fig). Despite the increased number of Th17 cells, the levels of IL-17 in the circulation of BXD2 mice were comparable to that of control mice (Fig. 2B). To further characterize the Th1 and Th17 cell responses, we analyzed the expression of Th1 and Th17-associated genes by using quantitative RT-PCR. As depicted in Fig. 2C, we observed that the levels of *Il17a* and *Rorc* mRNA expression in the spleens of BXD2 mice were comparable to those of control mice, while the levels of *Tbx21* (encoding T-bet in mouse) and *Ifng* were increased in the BXD2 mice.

Commensal bacteria such as segmented filamentous bacteria (SFB) can modulate Th17 cell responses [35,36]. To test if any differences of gut microbiota between BXD2 and control mice impact Th17 responses, we co-housed BXD2 mice with control mice for 4 weeks. The frequencies of Th17 cells in both Peyer’s patches and mesenteric lymph nodes were comparable between the two groups (S1C Fig). These results together demonstrate that Th17 cell response in BXD2 mice did not significantly differ from control mice in steady state.

**Fig 3:**
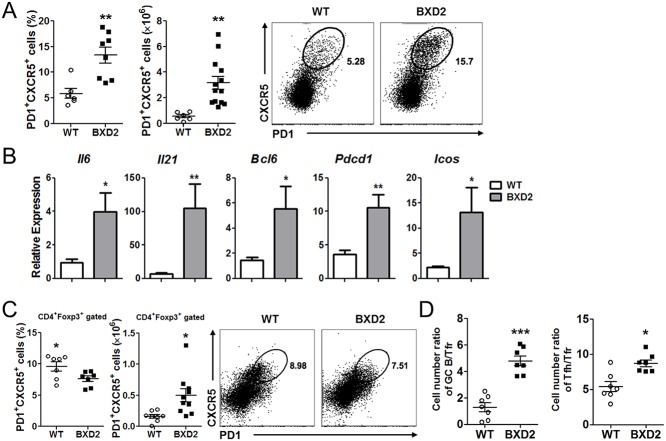
Tfh cell responses were increased in the BXD2 mice. (**A**) Flow cytometry analysis of the percentage and number of PD-1^+^CXCR5^+^ CD4^+^ T cells in the spleens of WT and BXD2 mice at the age of 6 months or older. (**B**) Quantitative RT-PCR analysis of Tfh cell related genes from WT and BXD2 mouse splenocytes at the age of 6 months or older. (C) Flow cytometry analysis of the percentage and number of CD4^+^Foxp3^+^PD-1^+^CXCR5^+^ Tfr cells in the spleens of WT and BXD2 mice at the age of 6 months or older. (D) Ratio of Foxp3^-^ Tfh cells to FoxP3^+^ Tfr cells in the mouse spleens and germinal center B cells to FoxP3^+^ Tfr cells in the mouse spleen. Data are represented as mean ± SEM. *p < 0.05, **p < 0.01, ***p < 0.001.

Since increased Tfh cells are associated with systemic autoimmune diseases [10,11,12], we next analyzed Tfh cell responses in the BXD2 mice. As shown in Fig. 3A, the frequency and number of PD1^+^CXCR5^+^ Tfh cells were significantly higher in the spleens of BXD2 mice compared to control mice. Moreover, the levels of Tfh cell signature genes, such as *Il6*, *Il21*, *Bcl6*, *Pdcd1* (encoding PD-1) and *Icos* were all significantly increased in the splenocytes of BXD2 mice compared with those in control mice (Fig. 3B). The frequency of Tfh cells in the other secondary lymphoid organs, except axillary lymph node and Peyer’s patches, remained comparable between BXD2 and wild-type mice (S1A Fig). Extrafollicular T helper cells are proposed to be involved in the development of antibody-mediated autoimmunity [37]. However, the frequency of PD-1^low^ extrafollicular T helper cells in BXD2 mice remained comparable to that of control mice (S2 Fig). Collectively, these results demonstrate that the spleens of BXD2 mice contained significantly increased Tfh cells and, to a lesser extent, Th1 cells, while the frequencies of Th17 cells and PD-1^low^ extrafollicular T helper cells were similar when compared with control mice.

### Analysis of Tfr cells in BXD2 mice

PD1^+^CXCR5^+^Foxp3^+^ Tfr cells have emerged as a specialized subset of regulatory T cells suppressing germinal center reactions *in vivo* [15,16,17]. Therefore, we determined if the frequency of Tfr cells as well as the ratio of Tfh cells and germinal center B cells per Tfr cell in the BXD2 mice differed from that of control mice. Although the frequency of total Foxp3^+^ T cells was found to be higher in BXD2 mice than control mice (S3 Fig), we observed that the frequency of Tfr cells was slightly but significantly lower in the spleens of the former group (Fig. 3C). The absolute number of Tfr cells, however, was higher in the spleens of BXD2 mice due to increased spleen cellularity (Fig. 3C). As shown in Figs. 1 and 3, the numbers of germinal centers, germinal center B cells and Tfh cells were all significantly increased in the BXD2 mice. Accordingly, the ratio of germinal center B cells per Tfr cell was significantly higher in BXD2 mice than control mice (Fig. 3D). Similarly, the ratio of Tfh cells per Tfr cell was also significantly higher in the former group (Fig. 3D).

### Correlation analysis between autoantibodies, germinal center B cells, Th17 and Tfh cells in BXD2 mice

**Fig 4:**
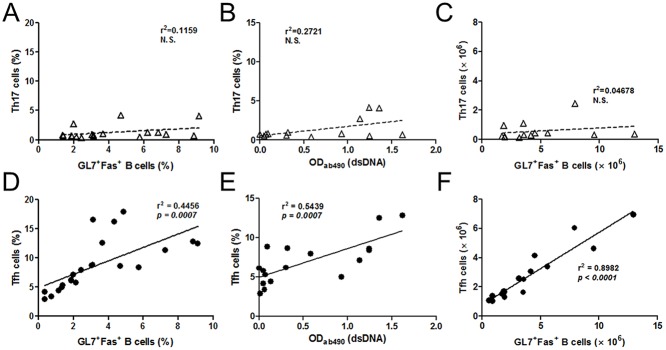
Positive correlation of Tfh cells, not Th17 cells with outputs of GC responses in BXD2 mice. Linear regression analysis of the frequency of Th17 cells (**A**) or Tfh cells (**D**) with that of germinal center B cells, and Th17 cells (**B**) or Tfh cells (**E**) with dsDNA specific autoantibody levels. Linear regression analysis of the number of Th17 cells (C) or Tfh cells (F) with that of germinal center B cells. Pearson correlation coefficients (r^2^) between the percent of T helper cell subset and of germinal center B cells or those of T helper cell subset and dsDNA specific autoantibodies levels are indicated in each graph.

Our results in Figs. 2 and 3 showed no evident increase in Th17 cell responses in BXD2 mice, which are contradictory to previous studies [30,31,34], suggesting that Th17 cell responses might not be associated with increased autoantibodies in the BXD2 mice. To address this possibility, we examined whether the levels of autoantibodies and the frequencies of germinal center B cells are correlated with the frequencies of Th17 or Tfh cells. Linear regression analysis showed no correlation between the frequencies of Th17 cells and those of germinal center B cells (Fig. 4A). Similarly, no correlation was observed between the frequencies of Th17 cells and the levels of anti-dsDNA (Fig. 4B) or between the numbers of Th17 cells and germinal center B cells (Fig. 4C). In sharp contrast, both the frequencies of germinal center B cells and the levels of anti-dsDNA showed a clear positive correlation with the frequencies of Tfh cells (Fig. 4D and 4E). Moreover, a strong correlation was observed between the number of Tfh cells and those of germinal center B cells (Fig. 4F). In addition, the frequencies of Th1 cells appeared to be, with a lesser extent, also positively correlated with those of germinal center B cells and the levels of anti-dsDNA (S4 Fig). As expected, the levels of anti-dsDNA showed strong positive correlation with the frequencies of germinal center B cells (S4D Fig), while the frequencies of Tfr cells showed no correlation with those of germinal center B cells (S4B Fig). Together, these linear regression analyses clearly demonstrate Tfh cell responses, but not Th17 cell responses, showed a strong positive correlation with auto-reactive germinal center B cell responses in the BXD2 mice.

### CXCR5^+^CD4^+^ T cells, but not CCR6^+^CD4^+^ T cells, from BXD2 mice induce IgG production from naïve B cells in an IL-21-dependent, IL-17-independent manner

**Fig 5:**
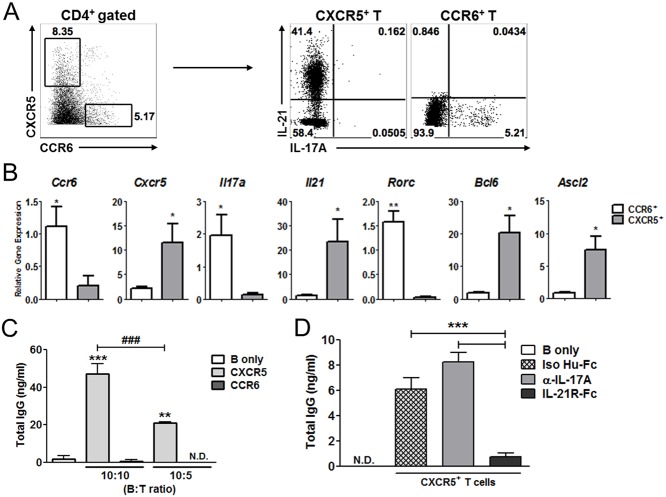
IL-21-producing CXCR5^+^CD4^+^ T cells of BXD2 mice, not IL-17-producing CCR6^+^CD4^+^ T cells, provide B cell help for IgG production. (**A**) CXCR5^+^ CD4^+^ T cells and CCR6^+^ CD4^+^ T cells were sorted and subjected to intracellular cytokine staining. (**B**) Quantitative RT-PCR analysis of Tfh or Th17 cell related gene expression in the CXCR5^+^ and CCR6^+^ CD4^+^ T cells from BXD2 mice (**C**) Naïve B cells (B220^+^IgD^+^GL7^-^) from BXD2 mice were co-cultured with CXCR5^+^ or CCR6^+^ CD4^+^ T cells from BXD2 mice for 7days. The total IgG levels were measured by ELISA (**D**) Different cytokine blocking reagents, isotype control antibody (Iso Hu-Fc), Rat anti mouse IL-17A antibody (α-IL-17A), or recombinant mouse IL-21 receptor Fc chimera (IL-21R-Fc) were added (10 μg/ml, every other day) into the cell culture described in (**C**) and the levels of total IgG were measured by ELISA. Data are represented as mean ± SEM. *p < 0.05, **p < 0.01, ***p < 0.001, ^###^p < 0.001 comparing 10:10–10:5 ratio of B:T co-culture condition.

Although previous studies described a critical contribution of Th17 cell responses to the generation of autoimmune B cell responses in BXD2 mice, our findings showed no evident correlation between Th17 cells and autoantibody responses. To further determine the contribution of Th17 cells and Tfh cells to the generation of spontaneous germinal center B cell responses in the BXD2 mice, we sought to determine whether Th17 cells and Tfh cells isolated from BXD2 mice can trigger IgG production from naïve B cells. Expression of CCR6 and CXCR5 on CD4^+^ T cells are reliable markers of Th17 and Tfh cells, respectively [2,38,39]. Hence, we purified CCR6^+^CD4^+^ T cells and CXCR5^+^CD4^+^ T cells from the spleens of 3–9 month-old BXD2 mice (Fig. 5A). When re-stimulated with these cells, purified CXCR5^+^ and CCR6^+^ cells almost exclusively expressed IL-21 and IL-17, respectively (Fig. 5A). Th17 cells are known to express IL-21 [40]; however, CCR6^+^CD4^+^ T cells from the BXD2 mice showed little expression of IL-21. Moreover, few CXCR5^+^CD4^+^ T cells from the BXD2 mice expressed IL-17 (Fig. 5A). Consistent with these results, quantitative RT-PCR analysis showed that the CXCR5^+^ population expressed significantly higher levels of *Il21*, *Bcl6* and *Ascl2* mRNA, while the CCR6^+^ population expressed higher levels of *Il17a* and *Rorc* (Fig. 5B). These results demonstrate the purified CCR6^+^ and CXCR5^+^ populations among the CD4^+^ T cells represented Th17 cells and Tfh cells in the BXD2 mice.

To determine the role of each CD4^+^ T cell subset from the BXD2 mice on the activation and differentiation of B cells, the purified T cells were cultured with B220^+^GL7^-^IgD^+^ naïve B cells isolated from BXD2 mice in the presence of anti-CD3 and anti-IgM for 7 days. As depicted in Fig. 5C, we found that CXCR5^+^CD4^+^ T cells efficiently stimulated B cells to produce IgG even when lower number of T cells was used (B:T cell ratio = 10:5). By contrast, CCR6^+^CD4^+^ T cells failed to do so, even when higher number of T cells was used (B:T cell ratio = 10:10) (Fig. 5C). To test the role of each helper T cell population on B cell expansion, we labeled B cells with CFSE before co-culturing them with T cells. Addition of CXCR5^+^ T cells induced significant B cell proliferation while CCR6^+^ T cells failed to do so (S5A Fig). Similarly, addition of Th17 cells differentiated *in vitro* from naïve BXD2 T cells did not induce IgG production from co-cultured naïve B cells in our experimental setting (S5B&C Fig). To determine the role of IL-17 and IL-21 in this process, anti-IL-17 or IL-21R-Fc fusion protein was added into the culture. As shown in Fig. 5D, addition of IL-21R-Fc almost completely abolished the production of IgG from naïve B cells co-cultured with CXCR5^+^CD4^+^ T cells of BXD2 mice, while addition of anti-IL-17 showed no effect. These results strongly suggest that CXCR5^+^CD4^+^ T cells in the BXD2 mice were responsible for the enhanced autoantibodies and germinal center B cell responses through an IL-21 dependent, IL-17-independent mechanism, while CCR6^+^CD4^+^ T cells played little role in the activation of auto-reactive B cells in BXD2 mice in our experimental setting.

## Discussion

Understanding the pathogenesis of autoimmunity is important not only for understanding the pathogenesis but also for the development of effective therapeutics for the treatment of the detrimental immune disorders in humans. However, the role of Th17 cells and Tfh cells in the development of antibody-mediated autoimmune diseases such as lupus and arthritis has been incompletely understood. In this study, we aimed to determine the correlation and contribution of Th17 and Tfh cells in the development of autoimmune B cell responses in the BXD2 mouse model of lupus. Our results demonstrate that Tfh cells are associated with increased autoimmune B cell responses, while Th17 cells play little role in this process because (i) there were no clear differences in the frequency of IL-17-producing Th17 cells, the level of IL-17 in circulation, or Th17-related gene expression between BXD2 mice and WT control mice, (ii) the frequency of Tfh cells, the ratio of Tfh cells per Tfr cell, and Tfh related gene expression were all increased in BXD2 mice compared to control mice, (iii) Tfh cells but not Th17 cells showed strong positive correlation with the frequency germinal center B cells and the levels of autoantibody to dsDNA, (iv) CXCR5^+^CD4^+^ T cells isolated from BXD2 mice stimulated naïve B cells to produce IgG, while CCR6^+^CD4^+^ T cells failed to do so, and (v) blockade of IL-21 but not Il-17 abolished the production of IgG induced by CXCR5^+^CD4^+^ T cells *in vitro*. Thus Tfh cells are responsible for the enhanced autoantibodies and germinal center B cell responses through an IL-21 dependent mechanism in BXD2 mice.

Strong correlation between increased Tfh responses and antibody-mediated systemic auto immunity, such as SLE, has been reported in both humans and experimental animals. Thus it seems reasonable to surmise that unnecessary aggressive Tfh cell responses are responsible for the onset of such antibody-mediated immune disorders [13,41,42]. In addition, SLE patients are known to exhibit increased levels of IL-17 in circulation when compared with healthy controls [43]. However, a recent study showed that the frequency of circulating Tfh cells rather than Th17 cells was correlated with disease activities in patients with SLE [11]. In addition, other studies argued that although serum levels of IL-17 were increased in SLE patients, there was no strong correlation between IL-17 levels and disease activity [44,45,46]. Therefore the role of IL-17/Th17 cells and auto-reactive B cell responses remains controversial.

The role of IL-17 in the auto-reactive autoimmune B cell responses has been well characterized in the BXD2 animal model of lupus [30,31,32]. The levels of IL-17 and the frequency of Th17 cells were shown to be elevated in the secondary lymphoid organs of BXD2 mice. The increased IL-17 was proposed to impact B cell chemotactic ability and stabilize the interaction of the germinal center B cells with nearby T cells [30,32]. IL-17 was also shown to facilitate the localization of Tfh cells in GC, but not the frequency and function of Tfh cells [31]. Nevertheless, our present study showed no evident association of Th17 cells and IL-17 in the generation of autoimmune B cell responses in BXD2 mice. For instance, the frequency of Th17 cells and the levels of IL-17 in circulation were comparable between BXD2 mice and control mice. Accordingly, Tfh cells but not Th17 cells showed a strong positive correlation with the levels of autoantibodies as well as the size of spontaneous germinal center B cell responses. By using a co-culture system, we clearly demonstrated that IL-21 rather than Il-17 was essential for triggering the production of IgG from naïve B cells. Therefore, our results support the idea that Tfh cells rather than Th17 cells are responsible for the development of auto-reactive B cell responses. This discrepancy on the role of Th17 cells in autoimmune B cell responses in BXD2 mice is not clear at the moment. One possible explanation is that differences in diet or gut microenvironment may result in different levels of Th17 responses in steady state. For instance, segmented filamentous bacteria (SFB) induce the generation of Th17 cells [35]. All mice in the present study were obtained from Jackson Laboratory, known to be SFB free [35], and maintained in a barrier facility. In addition, the frequency of Th17 cells in gut-associated lymphoid tissues of BXD2 mice was comparable to that of control mice even after 4 weeks of co-housing. Therefore the discrepancy of Th17 cell immunity between previous studies and our study is not likely because BXD2 mice harbored less Th17-inducing gut microbiota than control mice in the present study. Our finding strongly suggest that without increasing Th17- or IL-17 responses, autoimmune Tfh cell responses and auto-reactive B cell responses could be induced in BXD2 mice.

Tfr cells reside in B cell follicles and are known to be increased upon immunization with foreign protein antigen [15,16,17]. These cells inhibit germinal center reactions and production of isotype-switched immunoglobulin, although the mechanism of suppression is poorly understood. Interestingly, despite the fact that the frequencies of Tfh cells and germinal center B cells were increased in the BXD2 mice, the frequency of Tfr cells in the same mice appeared to be lower than that in control mice. As a consequence, the ratio of germinal center B cells per Tfr cell or Tfh cells per Tfr cell were all significantly increased in the BXD2 mice. This finding suggests that the suppression of germinal center B cell responses by Tfr cells is likely less efficient in the BXD2 mice, which might contribute to uncontrolled spontaneous germinal center reactions and subsequent development of a lupus phenotype in this strain.

We also observed increased IFN-γ-producing Th1 cells in BXD2 mice. Interestingly, *Sanroque* mice, which develop autoimmune lupus symptoms, exhibited increased IFN- γ. The enhanced IFN-γ was shown to trigger the accumulation of Tfh and GCs in this animal model [47]. Since IFN-γ has been known to be associated with autoimmune lupus, the increase of Th1 cells in BXD2 mice might play a role in the development of auto-reactive autoantibodies. Future studies will be needed to determine the role of Th1 responses in the generation of autoimmune germinal center B cell responses in animal models of lupus as well as in humans. Since IL-12 and IFN-γ can negatively regulate Th17 cell responses [48,49], it will be interesting to study the role increased Th1 responses in Th17 and Tfh cell responses as on the onset of autoimmunity in BXD2 mice.

Although IL-17 provides B cell help during GC reactions, many studies including our study, suggest that IL-21 has a direct role in GC responses and further induces B cell activation and differentiation. Currently, there are many on-going autoimmune disease clinical trials targeting IL-21 and its signaling pathway [50]. On the other hand, IL-17 has not yet been therapeutically targeted in human SLE or other related autoimmune diseases [43]. Therefore, our finding strongly supports that targeting Tfh cells or IL-21 rather than Th17 cells or IL-17 might be a better therapeutic approach. In summary, increase in Tfh cells rather than Th17 cells is correlated with auto-reactive autoimmune disease in BXD2 mice, and thus targeting Tfh cells, such as blocking IL-21, rather than Th17 cells, would be beneficial for the treatment of antibody-mediated autoimmune diseases in humans.

## Supporting Information

S1 Table Real time PCR primers used in this study. (PDF)

S1 Fig CD4^+^ T cell phenotypes in the secondary lymphoid organs of BXD2 mice. (**A**) Flow cytometric analysis of GL7^+^Fas^+^ GC B cells, PD-1^+^CXCR5^+^ CD4^+^ T cells, CD4^+^Foxp3^+^PD-1^+^CXCR5^+^ Tfr cells, IFN-γ^+^ Th1 or IL-17A^+^ Th17 cells in the indicated lymphoid organs from WT and BXD2 mice. (iLN: inguinal lymph node, aLN: axillary lymph node, mLN: mesenteric lymph node, PP: Peyer’s patch) (**B**) Frequency of IFN-γ^+^ or IL-17A^+^ cells in whole splenocytes of WT and BXD2 mice. (**C**) Percentage of Th17 cells in the mesenteric lymph nodes and Peyer’s patches from co-housed WT and BXD2 mice. Data are represented as mean ± SEM. *p < 0.05, **p < 0.01, ***p < 0.001.
(TIF)

S2 Fig Analysis of PD-1^low^ extrafollicular T helper cells in BXD2 mice. (**A**) Flow cytometric analysis of PD-1^low^CXCR5^+^ CD4^+^ T cells in the spleens of WT and BXD2 mice at the age of 3 months. (**B**) Flow cytometric analysis of PD-1^low^CXCR4^+^CXCR5^-^ CD4^+^ T cells in the spleens of WT and BXD2 mice at the age of 3 to 4 months. Data are represented as mean ± SEM. *p < 0.05, **p < 0.01, ***p < 0.001.
(TIF)

S3 Fig Analysis of Foxp3^+^ T cells in BXD2 mice. The percentage and absolute number of Foxp3^+^ CD4^+^ T cells in the spleens of WT and BXD2 mice. Data are represented as mean ± SEM. *p < 0.05, **p < 0.01, ***p < 0.001.
(TIF)

S4 Fig Linear regression analysis between Th1/Tfr cells and germinal center B cells. Linear regression analysis of the frequency of Th1 cells with GC B cells (**A**), the frequency of Tfr cells with germinal center B cells (**B**), Th1 cells with dsDNA specific autoantibody levels (**C**), Linear regression analysis of germinal center B cells with dsDNA specific autoantibody levels (**D**). Pearson correlation coefficients (r^2^) between the percent of T indicated helper T cell subset and of germinal center B cells or those of indicated T cell subset and dsDNA specific autoantibodies levels are indicated at each graph.
(TIF)

S5 Fig CXCR5^+^CD4^+^ T cells of BXD2 mice, not CCR6^+^CD4^+^ T nor *in vitro* differentiated Th17 cells, provide B cell help for IgG production. (**A**) Proliferation of CFSE labeled naïve B cells (B220^+^IgD^+^GL7^-^) from BXD2 mice obtained from the co-cultured with CXCR5^+^ or CCR6^+^ CD4 T cells from BXD2 mice for 7days. (**B**) Cytokines expression in *in vitro* differentiated Th17 cells 5 days after stimulation from naïve (CD4^+^CD25^-^CD44^-^CD62L^+^) CD4 T cells of BXD2 mice. (**C**) Naïve B cells (B220^+^IgD^+^GL7^-^) from BXD2 were co-cultured with *in vitro* differentiated Th17 cells described in (**B**) for 7 days and the levels of total IgG were measured by ELISA. Data are represented as mean ± SEM. *p < 0.05, **p < 0.01, ***p < 0.001.
(TIF)
