# Supplementary material for: Regulation of Autoimmune Germinal Center Reactions in Lupus-Prone BXD2 Mice by Follicular Helper T Cells
Source: PLoS One. 2015 Mar 13;10(3):e0120294. doi: 10.1371/journal.pone.0120294 (PMC4358919; doi:10.1371/journal.pone.0120294)
Supplement: S1 Table — (PDF) [file pone.0120294.s001.pdf]

Gene	5’ Primer	3’Primer
Ifng	GAT GCA TTC ATG AGT ATT GCC AAG T	GTG GAC CAC TCG GAT GAG CTC
Il17	CTC CAG AAG GCC CTC AGA CTA C	GGG TCT TCA TTG CGG TGG
Il6	TAT GAA GTT CCT CTC TGC AAG AGA	TAG GGA AGG CCG TGG TT
Il4	AGA TCA CGG CAT TTT GAA CG	TTT GGC ACA TCC ATC TCC G
Il21	TCA TCA TTG ACC TCG TGG CCC	ATC GTA CTT CTC CAC TTG CAA TCC C
Tbx21	CAA CAA CCC CTT TGC CAA AG	TCC CCC AAG CAG TTG ACA GT
Rorc	CCG CTG AGA GGG CTT CAC	TGC AGG AGT AGG CCA CAT TAC A
Bcl6	AGG CCT CCT TCC GCT ACA AG	CAA ATG TTA CAG CGA TAG GGT TTC T
Icos	CGG CAG TCA ACA CAA ACA A	TCA GGG GAA CTA GTC CAT GC
Pdcd1	CCG CCT TCT GTA ATG GTT TGA	GGG CAG CTG TAT GAT CTG GAA
Cxcr5	ACT CCT TAC CAC AGT GCA CCT	GGA AAC GGG AGG TGA ACC A
Ccr6	CCT CAC ATT CTT AGG ACT GGA GC	GGC AAT CAG AGC TCT CGG A
Ascl2	CGC TGC CCA GAC TCA TGC CC	GCT TTA CGC GGT TGC GCT CG
Id3	TGC TAC GAG GCG GTG TGC TG	AGT GAG CTC AGC TGT CTG GAT CGG
Itga4	AAT GCC TCA GTG GTC AAT CC	CTA CCC AGC TGG AGC TGT TC
Itgb2	CCG ACA ACT CCA ACC AGT TT	AGC AGC CTC GTG ACA TTG CGC
Actb	TGG AAT CCT GTG GCA TCC ATG AAA C	TAA AAC GCA GCT CAG TAA CAG TCC G
