# Supplementary figures and images for: Regulation of Autoimmune Germinal Center Reactions in Lupus-Prone BXD2 Mice by Follicular Helper T Cells
Source: PLoS One. 2015 Mar 13;10(3):e0120294. doi: 10.1371/journal.pone.0120294 (PMC4358919; doi:10.1371/journal.pone.0120294)

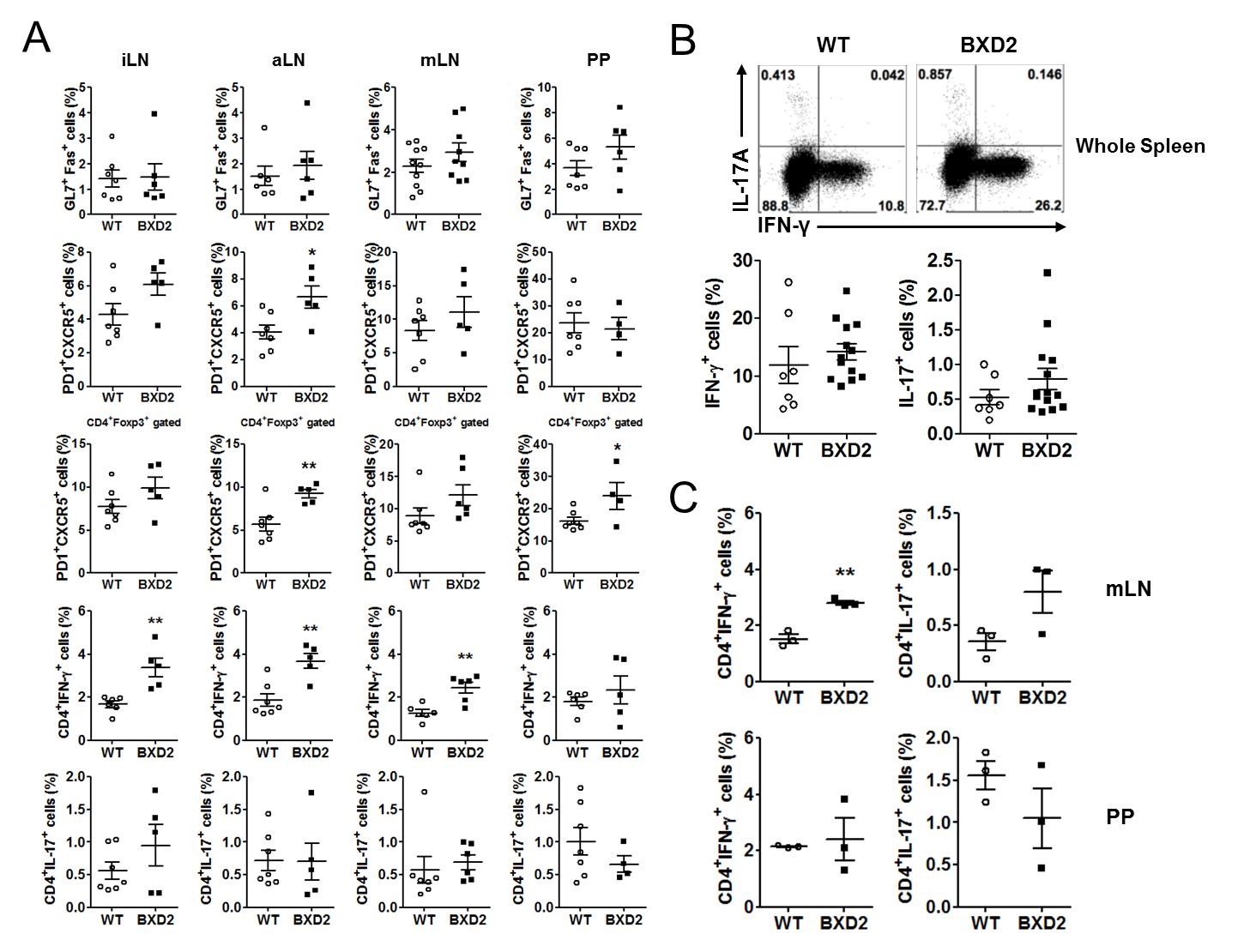

Supplement: S1 Fig — (A) Flow cytometric analysis of GL7+Fas+ GC B cells, PD-1+CXCR5+ CD4+ T cells, CD4+Foxp3+PD-1+CXCR5+ Tfr cells, IFN-γ+ Th1 or IL-17A+ Th17 cells in the indicated lymphoid organs from WT and BXD2 mice. (iLN: inguinal lymph node, aLN: axillary lymph node, mLN: mesenteric lymph node, PP: Peyer’s patch) (B) Frequency of IFN-γ+ or IL-17A+ cells in whole splenocytes of WT and BXD2 mice. (C) Percentage of Th17 cells in the mesenteric lymph nodes and Peyer’s patches from co-housed WT and BXD2 mice. Data are represented as mean ± SEM. *p < 0.05, **p < 0.01, ***p < 0.001. (TIF) [file pone.0120294.s002.tif]

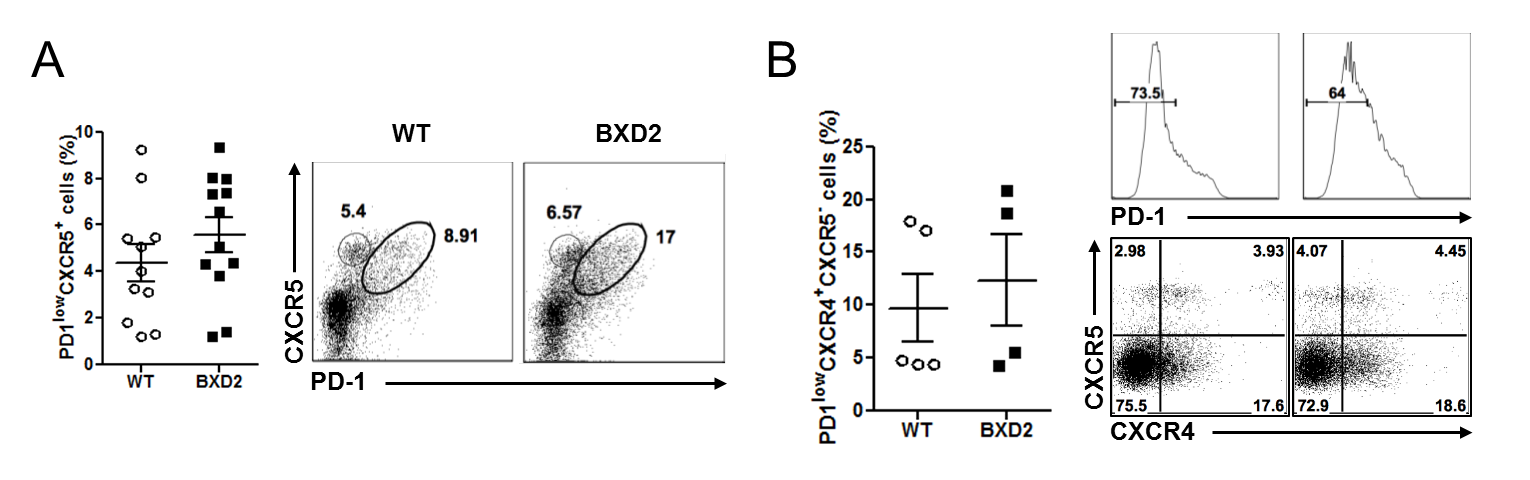

Supplement: S2 Fig — (A) Flow cytometric analysis of PD-1lowCXCR5+ CD4+ T cells in the spleens of WT and BXD2 mice at the age of 3 months. (B) Flow cytometric analysis of PD-1lowCXCR4+CXCR5- CD4+ T cells in the spleens of WT and BXD2 mice at the age of 3 to 4 months. Data are represented as mean ± SEM. *p < 0.05, **p < 0.01, ***p < 0.001. (TIF) [file pone.0120294.s003.tif]

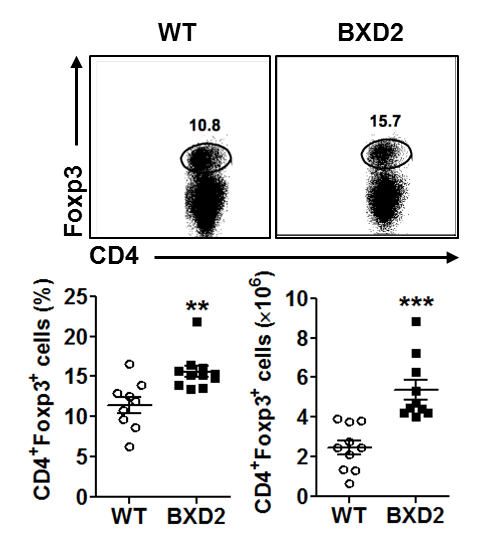

Supplement: S3 Fig — The percentage and absolute number of Foxp3+ CD4+ T cells in the spleens of WT and BXD2 mice. Data are represented as mean ± SEM. *p < 0.05, **p < 0.01, ***p < 0.001. (TIF) [file pone.0120294.s004.tif]

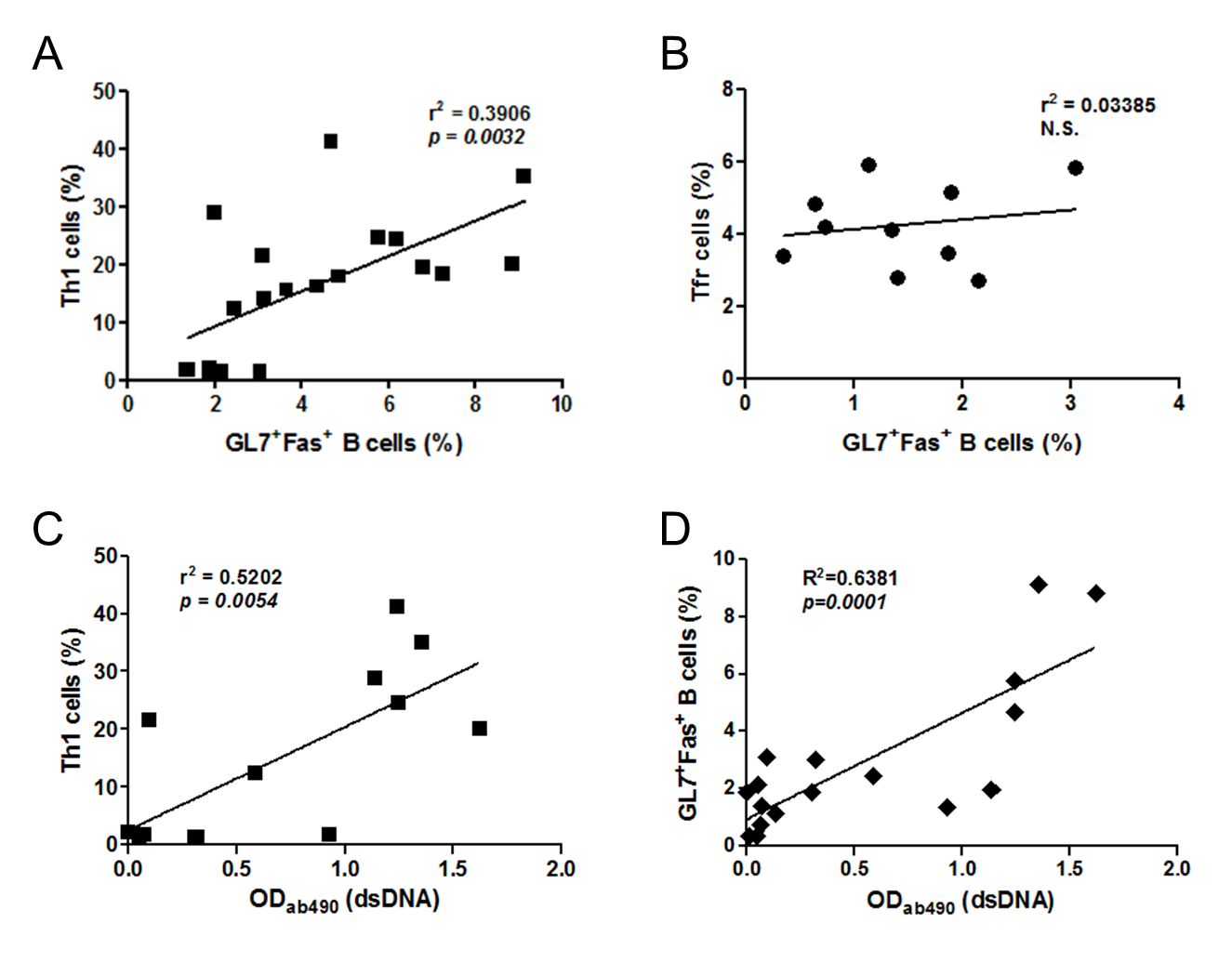

Supplement: S4 Fig — Linear regression analysis of the frequency of Th1 cells with GC B cells (A), the frequency of Tfr cells with germinal center B cells (B), Th1 cells with dsDNA specific autoantibody levels (C), Linear regression analysis of germinal center B cells with dsDNA specific autoantibody levels (D). Pearson correlation coefficients (r2) between the percent of T indicated helper T cell subset and of germinal center B cells or those of indicated T cell subset and dsDNA specific autoantibodies levels are indicated at each graph. (TIF) [file pone.0120294.s005.tif]

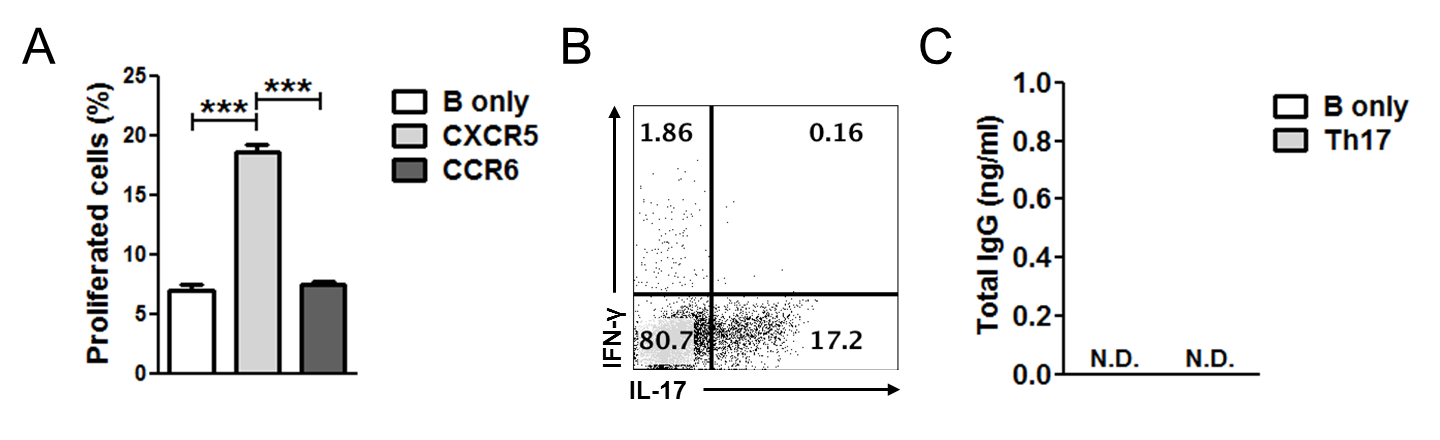

Supplement: S5 Fig — (A) Proliferation of CFSE labeled naïve B cells (B220+IgD+GL7-) from BXD2 mice obtained from the co-cultured with CXCR5+ or CCR6+ CD4 T cells from BXD2 mice for 7days. (B) Cytokines expression in in vitro differentiated Th17 cells 5 days after stimulation from naïve (CD4+CD25-CD44-CD62L+) CD4 T cells of BXD2 mice. (C) Naïve B cells (B220+IgD+GL7-) from BXD2 were co-cultured with in vitro differentiated Th17 cells described in (B) for 7 days and the levels of total IgG were measured by ELISA. Data are represented as mean ± SEM. *p < 0.05, **p < 0.01, ***p < 0.001. (TIF) [file pone.0120294.s006.tif]
